# A Model of Ischemia-Induced Neuroblast Activation in the Adult Subventricular Zone

**DOI:** 10.1371/journal.pone.0005278

**Published:** 2009-04-23

**Authors:** Davide Vergni, Filippo Castiglione, Maya Briani, Silvia Middei, Elena Alberdi, Klaus G. Reymann, Roberto Natalini, Cinzia Volonté, Carlos Matute, Fabio Cavaliere

**Affiliations:** 1 IAC, CNR, Rome, Italy; 2 CNR Institute of Neuroscience, Santa Lucia Foundation, Rome, Italy; 3 Neurotek-UPV/EHU, Department of Neuroscience and CIBERNED, Zamudio, Spain; 4 IfN-Leibniz Institute for Neurobiology, Magdeburg, Germany; 5 CNR Institute of Neurobiology and Molecular Medicine, Santa Lucia Foundation, Rome, Italy; Chiba University Center for Forensic Mental Health, Japan

## Abstract

We have developed a rat brain organotypic culture model, in which tissue slices contain cortex-subventricular zone-striatum regions, to model neuroblast activity in response to in vitro ischemia. Neuroblast activation has been described in terms of two main parameters, proliferation and migration from the subventricular zone into the injured cortex. We observed distinct phases of neuroblast activation as is known to occur after in vivo ischemia. Thus, immediately after oxygen/glucose deprivation (6–24 hours), neuroblasts reduce their proliferative and migratory activity, whereas, at longer time points after the insult (2 to 5 days), they start to proliferate and migrate into the damaged cortex. Antagonism of ionotropic receptors for extracellular ATP during and after the insult unmasks an early activation of neuroblasts in the subventricular zone, which responded with a rapid and intense migration of neuroblasts into the damaged cortex (within 24 hours). The process is further enhanced by elevating the production of the chemoattractant SDf-1α and may also be boosted by blocking the activation of microglia. This organotypic model which we have developed is an excellent in vitro system to study neurogenesis after ischemia and other neurodegenerative diseases. Its application has revealed a SOS response to oxygen/glucose deprivation, which is inhibited by unfavorable conditions due to the ischemic environment. Finally, experimental quantifications have allowed us to elaborate a mathematical model to describe neuroblast activation and to develop a computer simulation which should have promising applications for the screening of drug candidates for novel therapies of ischemia-related pathologies.

## Introduction

### Adult neurogenesis

Generation of newborn neurons in the adult mammalian brain occurs throughout life in specific neurogenic structures. The subgranular zone is located in the dentate gyrus of the hippocampus and generates newborn neurons. These migrate into the granule cell layer or the CA1 region under physiological and/or pathological conditions such as ischemia [1]–[3]. Recently the posterior periventricular zone (pPV) has also been reported to be a neurogenic region in the hippocampus [4], [5]. The pPV has been identified in organotypic cultures as a part of the lateral ventricle wall lining the *stratum oriens*. Neuronal precursors have been identified as nestin^+^ cells responding to basic fibroblast growth factor treatment. Also, the peripheral nervous system has regions which exhibit characteristics of neuroregeneration and cell proliferation, such as the carotid body [6]. This body acts as an oxygen-sensing organ which produces new neuron-like cells from glia-like sustentacular cells.

The best characterized region which sustains adult neurogenesis is the subventricular zone (SVZ). This is the region which contributes to the regeneration of interneurons in the olfactory bulb. Under certain pathological condition, the SVZ can also contribute to neurogenesis for damaged structures such as the cerebral cortex after cerebral ischemia or Alzheimer disease [7], [8] or the striatum in an animal model of Parkinson's disease [9].

### Structure of the subventricular zone

The SVZ is located close to the ependyma, the thin layer that lines the lateral ventricle, and exhibits a specific cellular structure and molecular milieu which constitutes an optimal niche for neural precursors. It has been suggested that ependymal cells are the adult neural stem cells (NSCs) responsible for neurogenesis in the adult brain [10]. However, it is also claimed that NSCs are derived from the *type B* cell subpopulation which expresses the astrocytic marker GFAP. These cells are morphologically and functionally different to mature astrocytes. Furthermore, more recently, it has been demonstrated that, among these GFAP^+^ cells, only a monociliated subset protruding cilia into the lateral ventricle are really responsible for neurogenesis [11]. *Type B* cells are in contact with *type C* cells (transient amplifying precursors) and both ensheathe *type A* cells, the migrating neuroblasts [12]. The identification of both subclasses of precursors, cell types *C* and *A*, was mainly based on morphological observation and by the expression of specific markers such as doublecortin (DCX) and polysialylated neural adhesion molecule (PSA-NCAM), both co-expressed during proliferation (indicated experimentally by bromodeoxyuridine-BrdU incorporation). However, the use of such molecular markers to identify neural precursors in vivo has not been demonstrated convincingly until recently. Therefore, the term “neural precursor” may be used to describe a dividing cell with the ability to differentiate into a neural or neuronal population [12]. Cells obtained from SVZ in culture can grow in neurospheres and proliferate in response to mitogens (mainly bFGF and EGF).

Under normal conditions, precursors generated in the SVZ migrate through the rostral migratory stream, “walking” on a cord mainly made by astrocytes. Migrating precursors still maintain their ability to proliferate and to incorporate BrdU, but once they enter the olfactory bulb, they start to differentiate.

### Neurogenesis under pathological conditions

Neurogenesis can occur in brain regions which are damaged due to pathological conditions such as ischemia or, more recently, multiple sclerosis [2], [13], [14] via similar mechanisms. In this case, the local environment influenced by cellular stress plays a crucial role in modulating the mechanisms of proliferation, migration and differentiation. For example, after focal ischemia, the damaged cortex and SVZ communicate with each other by emitting chemotactic messages which induce a neurogenic response from the SVZ into the cortex [15], [16]. Factors which can positively modulate neurogenesis include the release of chemokines, mainly SDf-1α or IFN**γ**, whereas in contrast, cytotoxic extracellular ATP levels (with consequent ionotropic P2X_1–7_ and metabotropic P2Y_1,2,4,6,11–14_ receptor hyperactivation), glutamate or IL1-6 release impede the neurogenic effect. Although an abundant number of stem cell studies have been carried out over the last decade, the mechanisms and functional implications of adult neurogenesis under both physiological and pathological conditions still needs to be investigated in more depth. The principal questions which remain to be addressed are: 1) does a common progenitor exist for the three mature neural lineages? 2) What are the mechanisms which impede activation of neurogenesis, survival and integration into the neural network and 3) can the activation of neurogenesis following brain insult be recapitulated in a general law?

Mathematical modelling and computer simulations play a steadily increasing role in the study of biological systems. Both aim at explaining complex physiological and pathological phenomena in terms of basic physical processes. Numerical modelling complements the traditional empirical and experimental approach of biomedical research since they are able to provide effective ways to organize existing data. In addition, they focus experiments through hypothesis generation, identify critical areas where data are missing, and allow virtual experimentation whenever real experiments are impractical or just too expensive.

In this paper, we wish to address the latter two queries. We have studied the proliferative and migratory behavior of neuroblasts activated by oxygen/glucose deprivation (OGD) in an organotypic model which includes the neurogenic SVZ and the cortex. This has allowed us to localize biological factors which modulate neurogenesis under pathological conditions and to generate a mathematical analysis for neuroblast behavior on the basis of biological observations. In particular, we have analyzed attractant (SDf-1α) and repellent (extracellular ATP and microglia inflammation) factors which modulate neuroblast recruitment.

## Materials and Methods

### Cell cultures

All experiments were conducted under the supervision and with the approval of our internal animal ethics committee (Neurotek-UPV/EHU). Animals were handled in accordance with the European Communities Council Directive. All possible efforts were made to minimize animal suffering and the number of animals used.

#### Organotypic cultures

Cultures were prepared from coronal cerebral sections (400 µm of thickness) of brains from Wistar rat pups (2–3 days old) [16] using a modification of the method by Plenz and Kitai [17]. The brain was sliced in such a way as to maintain the connection between the subventricular zone and corpus callosum. Slices were plated on Millicell CM culture inserts (Millipore, Schwalbach, Germany) and maintained in 75% HME 03 (Cell concept, Berlin, Germany), 2 mM L-glutamine (Biochrom, Berlin, Germany), 25% horse serum (Gibco, Eggenstein, Germany) and 25 mg/ml gentamycin (Biochrom) for 3 days at 37°C, and then shifted to 33°C in Neurobasal medium supplemented with 0.5% B27 supplement (both from Gibco). More detailed information about organotypic culture preparation can be found supplementary information (Text S1).

#### Primary neurospheres from the SVZ

The SVZ region of Wistar rat pup brains (2–3 days old) was dissected under microscope and diced with a McIlwain Tissue Chopper (Campden Instruments, Pasadena, USA). Tissue was incubated for 7 minutes at 37°C with 0.5 g/l trypsin and 0.2 g/l EDTA (both from Sigma, Madrid, Spain). Trypsin was then inhibited by the addition of trypsin inhibitor and 0.001% DNAseI (Sigma) for 2 minutes at room temperature. Tissue was collected by centrifugation, washed 3 times with PBS and triturated with glass Pasteur pipette and 1 ml plastic tip. The cell suspension was resuspended in Neurobasal medium (Gibco, Barcelona, Spain) supplemented with 0.5% B27 supplement, 0.7% w/v glucose, 10 mM glutamine, 20 ng/ml EGF, 10 ng/ml bFGF and 20 ng/ml PEDF (all from Promega, Madrid, Spain) and plated at a density of 4×10^5^ cells/cm^2^. Medium (50%) with growth factors was changed every 2 days.

#### N9 cell line

The mouse N9 microglial cell line [18] was cultivated at 37°C in Iscove's modified Dulbecco's medium (IMDM, ICN, Eschwege, Germany) containing 5% Fetal Calf Serum (Gibco), 2 mM glutamine, penicillin (100 IU/ml), streptomycin (100 µg/ml) and 50 µM ß-mercaptoethanol.

### Oxygen/glucose deprivation

Glucose-Free Medium (GFM; 120 mM NaCl, 4 mM KCl, 2 mM MgSO_4_, 2 mM CaCl_2_, 2 mM KH_2_PO_4_ and 2 mg/ml mannitol, pH 7.4) was saturated with 95% N_2_. After saturation, the inserts with organotypic slices or the N9 cell line were placed in 1 ml saturated GFM and then maintained at 37°C for 30 min in an N_2_ saturated environment. For control conditions, medium consisted of GFM supplemented with 1 mg/ml glucose instead of mannitol. GFM was then replaced with Neurobasal medium and the cultures were kept under normoxic conditions for different periods of time at 33°C before evaluating cell death.

### Immunofluorescence

Treated slices were fixed for 40 min in 4% paraformaldehyde and saturated at room temperature in 1% BSA in PBS containing 0.5% Tween. The slices were then incubated overnight at 4°C with different primary antisera in 1% BSA in PBS (see Table 1 for specific working concentrations of primary and secondary antibodies). After further washing, the cultures were incubated in a solution containing a mixture of the secondary antibodies. After final washing, the plates were coverslipped with anti-fading gel/mount (Biomeda, CA, USA). Immunofluorescence was visualized by a scanning confocal microscope (LSM 510, Zeiss) equipped with an argon laser emitting at 488 nm and a helium/neon laser emitting at 543 nm.

**Table 1:** List of antibodies used for fluorescence immunochemistry.

Antibody	Work. dilution	Host	Company
**Primary**			
Double cortin (DCX)	1/200	Mouse	Santa Cruz, Milan-Italy
Bromo de-oxy uridine	1/200	Mouse	Sigma, Milan-Italy
Vinculin	1/200	Rabbit	AbCam, Milan-Italy
P2X1	1/500	Rabbit	Alomone, Jerusalem-Israel
P2X2	1/200	Rabbit	Alomone, Jerusalem-Israel
P2X3	1/500	Rabbit	Alomone, Jerusalem-Israel
P2X4	1/100	Rabbit	Alomone, Jerusalem-Israel
P2X5	1/200	Rabbit	Alomone, Jerusalem-Israel
P2X6	1/200	Rabbit	Alomone, Jerusalem-Israel
P2X7	1/500	Rabbit	Alomone, Jerusalem-Israel
CXCR4	1/200	Rabbit	Santa Cruz, Milan-Italy
**Secondary**			
Cy2 conj. Anti rabbit	1/200	Donkey	Dianova, Hamburg-Germany
Cy3 conj. Anti mouse	1/200	Donkey	Dianova, Hamburg-Germany
Cy3 conj. Anti goat	1/200	Donkey	Dianova, Hamburg-Germany

### Cell damage

Cell death in organotypic cultures was evaluated by cellular uptake of propidium iodide (PI). Cultures were incubated with culture medium containing PI (10 µM) for 2 h at 37°C. Afterwards, the slices were excited with a 510–560 nm light and the emitted fluorescence acquired at 610 nm using a rhodamine filter on an inverted fluorescence microscope (Eclipse TE 300, Nikon). Images were taken using a CCD camera and analyzed on a PC with image analysis software (LUCIA, Nikon). The uptake of PI to identify degenerating cells has been performed accordingly to Pozzo Miller [19].

### Cell proliferation

Cells were incubated for two hours with 20 µM bromodeoxyuridine (BrdU), which is incorporated into proliferating cells. In order to label different phases of proliferation, slices were fixed 24 or 120 hours after OGD. Immunofluorescent detection of BrdU was performed as described above (see Table 1 for antibody concentrations).

### Cell migration

#### YFP transgenic mice

Organotypic co-culture experiments were performed with SVZ from transgenic B6.Cg-TgN(Thy1-YFP)16Jr mice (The Jackson Laboratory; distributed by Charles River). As published elsewhere [20], these transgenic mice express yellow florescent protein (EYFP) at an enhanced level in subset of central neurons. The SVZ from YFP mice was co-cultivated in contact with wild type mouse cortex (see organotypic preparation above). YFP fluorescent cells migrating from the SVZ were detected in the cortex by fluorescent detection 24 hours after OGD.

#### Neuronal tracking

Wistar rat pups (4 days old) were anesthetized by trichloroacetaldehyde hydrate. The neuronal tracer Vybrant DiO (20 µl; Molecular Probes, Mi, Italy) was injected into the ventricle (i.v. injection) (coordinates: 1.4 mm posterior to the Bregma; 1.8 mm lateral to the midline; 3.5 mm from the brain surface). After 8 hours, co-cultures of labeled SVZ and contralateral (unlabeled) cortex were prepared. OGD experiments were performed after 10 DIV and green migrating cells were localized 24 hours later in the contralateral cortex.

#### Morphological analysis

Neuroblast migration was detected by morphological observation after double immunofluorescence for DCX and vinculin, both used at a concentration of (1∶200). Migrating cells were identified as amoeboid-shaped bodies revealed by vinculin expression.

### Measurement of [Ca^2+^]_i_

Whole neurospheres at 7 DIV were attached to poly-ornithine coated coverslips and loaded with fura-2 AM (5 µM; Invitrogen, Carlsbad, CA) in Neurobasal/B27 medium for 45 min at 37°C. Cells were washed in HBSS containing 20 mM HEPES, pH 7.4, 10 mM glucose and 2 mM CaCl_2_ (incubation buffer) for 5 min at room temperature. Experiments were performed in a coverslip chamber continuously perfused with incubation buffer at 1 ml/min. The perfusion chamber was mounted on the stage of a Zeiss (Oberkochen, Germany) inverted epifluorescence microscope (Axiovert 35), equipped with a 150 W Polychrome IV xenon lamp (T.I.L.L. Photonics, Martinsried, Germany) and a Plan Neofluar 40× oil immersion objective (Zeiss). Single cells within neurospheres were selected with a high-resolution digital black/white CCD camera (ORCA; Hamamatsu Photonics Iberica, Barcelona, Spain), and image acquisition and data analysis were performed using the AquaCosmos software program (Hamamatsu Photonics Iberica). [Ca^2+^]_i_ was estimated by the 340/380 ratio method, using a *K*_d_ value of 224 nM. At the end of the assay, *in situ* calibration was performed with the successive addition of 10 mM ionomycin and 2 M Tris/50 mM EGTA, pH 8.5. Data were analyzed with Excel (Microsoft, Seattle, WA) and Prism (GraphPad Software, San Diego, CA) software.

### ELISA

Organotypic cultures at 10 DIV were deprived of oxygen and glucose for 30 minutes as described above. Medium was collected at different times after OGD and subjected to ELISA, using the commercial kit Quantikine® for SDf-1α (R&D system, Milan, Italy). Positive reactions were evaluated using a Multiscan EX ELISA reader (Labsystems, Orlando, USA).

### Statistical analysis

Values were normalized as specified in each Figure legend. Statistical differences were evaluated by one-way analysis of variance (ANOVA), followed by *post hoc* test (HSD Tukey). All values reported, where *n* is the number of experiments, are significant, with p<0.05.

## Results

### Characterization of organotypic cultures as a model to study adult neurogenesis

In this paper, we use rat organotypic culture from cortex/SVZ/striatum to model neuroblast activation after brain ischemia. The presence of the SVZ in the slices, and its connectivity to the striatum and cortex through the *corpus callosum*, confers a 3D structure which facilitates an examination of the mechanisms of adult stem cell proliferation and neurogenesis. As shown in Figure S1, these cultures contain a region originally flanking the lateral ventricle, of high proliferative activity, as assessed by BrdU incorporation, with an approximate area of 0.9 mm^2^; we assume this to be equivalent to the SVZ. We used doublecortin (DCX) and BrdU as markers for neuroblasts [21] and proliferation [22] respectively. Confocal analysis after double immunofluorescence for BrdU and DCX revealed a high concentration of proliferating neuroblasts (approximately 5000 neuroblasts/mm^2^) in the SVZ (Figure S1). We also observed ciliated cell activity with microfluxes in the space surrounding the SVZ (see Video S1) indicating that the organotypic structure recapitulates the SVZ with its ciliated ependymal cells. Other structures, such as the cortex, maintain neuronal viability and substructure as demonstrated by lentivirus-mediated GFP infection (Figure S2).

### OGD induced neuroblast activation

**Figure 1:**
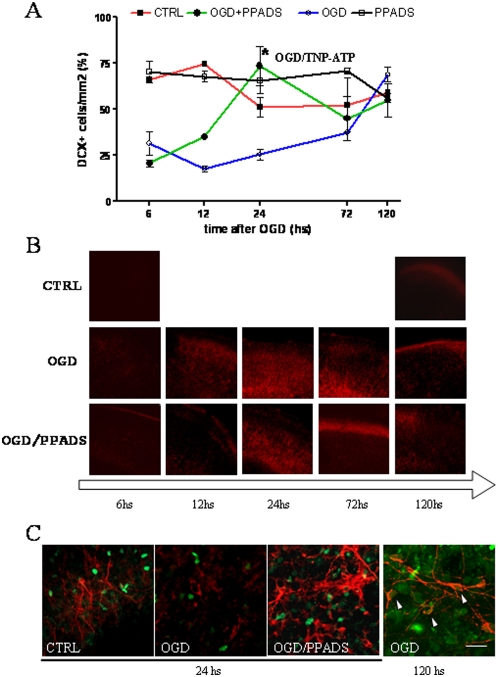
Pharmacological blocking of P2X ATP receptors during OGD increases the number of neuroblasts in the damaged cortex. Cortex/SVZ/striatum organotypic cultures at 10 DIV were subjected to 30 minutes of OGD. PPADS (100 µM) when used (OGD/PPADS) was added during, and for different times after OGD. (A) All neuroblasts in the cortex were counted at different times after OGD and expressed as the number of DCX expressing cells per mm2. Neuroblasts were also counted 24 hours after OGD in cultures treated with 50 µM TNP-ATP (asterisk). Counts represent means±SEM (n = 6). Control cultures were maintained in the presence of 1 mg/ml glucose. (B) Cell damage in the cortex was visualized by PI incorporation at different time points after OGD in the presence or absence of PPADS. (C) Organotypic cultures were subjected to OGD in the presence or absence of 100 µM PPADS. BrdU (20 µM) was added to the cultures 2 hours before fixing the slices and slices were maintained in culture for 24 hours or 5 days after OGD. Immunofluorescence was performed with anti-DCX (red) and anti-BrdU (green) antibodies. The control condition was conducted in the presence of 1 mg/ml glucose. Arrows indicate co-localization. The scale bar represents 20 µm.

To model neuroblast activity, we quantified proliferation and migration from the SVZ into the cortex as experimental parameters. We subjected organotypic cultures (at 10 DIV) to 30 minutes of OGD [16], [23] and counted the number of neuroblasts (measured as DCX^+^ cells) in the cortex, at different times after the insult. Counts showed early and drastic decreases within the first 6–12 hours, with a tendency to recover to normal levels 5 days after the insult (Figure 1A). Direct counting of DCX^+^ cells revealed 30, 18 and 25 neuroblasts/mm^2^ in the cortex at 6, 12 and 24 hours respectively after the insult and 69 at the end of the experiment (5 days after OGD).

On the basis of these observations, we hypothesized that the early decrease in neuroblast number during the first 12 hours might be due to metabolic stress induced by OGD which leaves neuroblasts and stem cells in the SVZ in a quiescent state. Our previous results had demonstrated that extracellular ATP induces excitotoxicity after metabolism impairment and that the blocking of P2X receptors protects against neuronal death [24]–[26]. We tested the effect of PPADS, a broad spectrum antagonist for P2X receptors [27], on the presence of DCX^+^ cells in the cortex. PPADS was applied at 100 µM during and after OGD and the number of DCX^+^ neuroblasts in the cortex was counted at different times after the insult. PPADS was ineffective 6 hours after OGD but the number of DCX^+^ cells started to increase 12 hours later, reaching a maximal level at 24 hours (73 neuroblasts/mm^2^) (Figure 1A). It should be noted that after PPADS administration, the number of DCX^+^ cells slightly decreased to 45 and 55 neuroblasts/mm^2^ at 3 and 5 days post OGD, which are levels similar to those found in control and OGD cultures. However, this falloff may be simply due to the ageing of the cultures. Another P2X receptor antagonist, trinitrophenyl adenosine triphosphate (TNP-ATP), was tested at the point of maximal effect of P2X blockade and it showed the same results (Figure 1A). As demonstrated by PI incorporation, PPADS was also effective in protecting against cortical cell damage (Figure 1B) with a maximum effect at 24 hours (as demonstrated by untreated/treated PI incorporation ratio, data not shown).

In order to study the effect of PPADS on neuroblast proliferation, cultures were primed with 20 µM BrdU and further analyzed after OGD. These BrdU experiments showed an “early” and “late” neuroblast population. The early population, counted in the cortex as the number of DCX^+^ cells during the first 24 hours, was sustained by PPADS treatment and did not show BrdU labeling (Figure 1C). The late population was observed in the cortex 5 days after OGD, in PPADS treated and untreated slices, and was also positive for BrdU staining. This finding indicates that the early neuroblast population migrated from the SVZ without proliferating.

**Figure 2 pone-0005278-g002:**
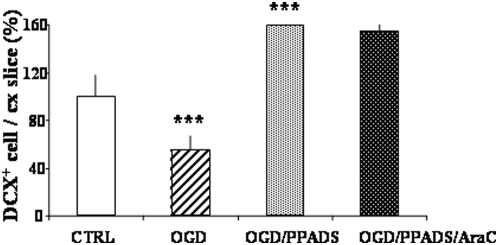
DCX expressing cells counted in the cortex do not proliferate. Cortex/SVZ/striatum organotypic cultures at 10 DIV were subjected to 30 minutes of OGD under different experimental conditions. PPADS (100 µM) and the mitotic inhibitor AraC (20 µM) were added during and after OGD. Cultures were fixed 24 hours later and subsequently labeled for DCX expression. Labeled cells in the cortex were counted as a percentage with respect to the control condition. Counts represent means±SEM (n = 4).

The antimitotic agent AraC was used together with PPADS during OGD to confirm that neuroblasts observed in the cortex 24 hours after the insult were not proliferating (Figure 2). After OGD, DCX^+^ cells counted in the cortex decreased by 45–50% with respect to the control, whereas antagonism of P2X receptors by PPADS restored the number of neuroblasts to 150–160% of control values. The inhibition of proliferation by AraC did not affect the number of neuroblasts in the cortex, thus confirming that the increase in the number of neuroblasts induced by the blockade of P2X receptors was not due to a local proliferative event.

### Neuroblast migration from the SVZ to the cortex

**Figure 3:**
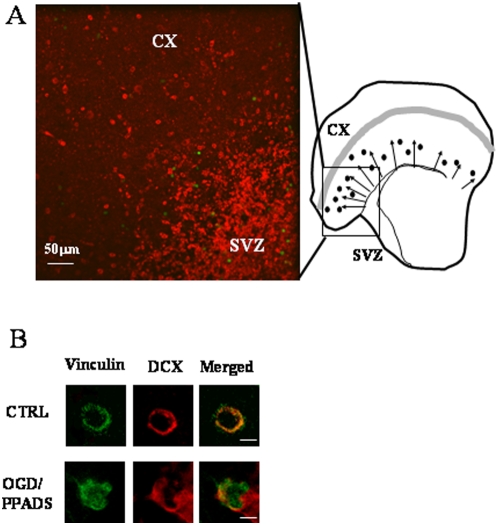
Neuroblasts migrate from the SVZ into the cortex. Morphological evidence. (A) Slices at 10 DIV were fixed 24 hours after OGD in the presence of 100 µM PPADS. The photo shows DCX immunofluorescence and was taken at the border between the SVZ-corpus callosum-cortex (cx) as depicted in the scheme to the right. (B) Slices were also double labeled with vinculin and DCX and photographs were taken in the cortex. Bar represents 10 µm.

Enhanced migration into the cortex induced by P2X receptor blockade during OGD was also suggested by cell spreading observed by DCX immunofluorescence 24 hours after PPADS/OGD treatment (Figure 3A). Neuroblasts (detected as DCX^+^ cells) were abundantly present in the SVZ and seemed to migrate into the cortex, since they exhibited a higher migratory density in the proximal in comparison to the distal part of the corpus callosum. Moreover, they exhibited an amoeboid-like morphology when double stained with DCX and vinculin, an intermediate cytoskeleton filament (Figure 3B). Migration of neuroblasts induced by P2X receptor pharmacological blockade was further demonstrated in experiments with co-cultures.

**Figure 4:**
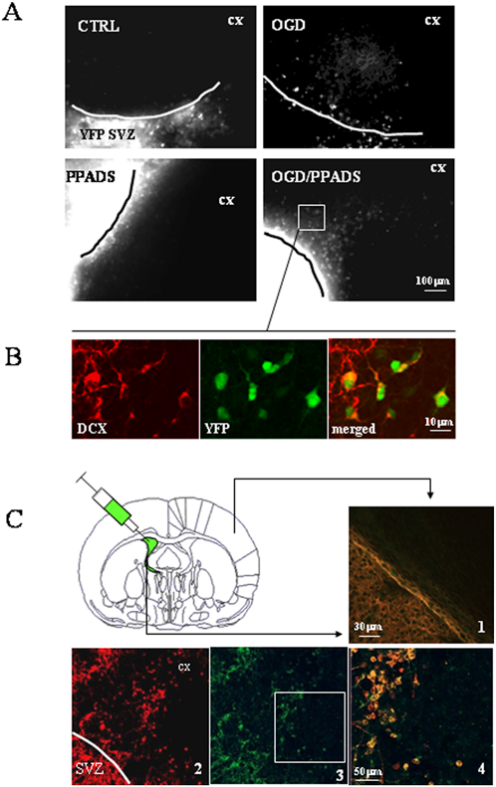
Neuroblasts migration from the SVZ into the cortex. Co-culture experiments. *YFP co-cultures*. (A) The SVZ from a 4 day-old YFP transgenic mouse was tightly placed in contact with cortex from a wild type mouse. OGD was performed and migration of fluorescent neuroblasts from the SVZ to the cortex (cx) was observed 24 later in the presence (OGD/PPADS) or absence (OGD) of 100 µM PPADS. Controls were performed in the presence of 1 mg/ml glucose (CTRL) and PPADS. (B) Co-cultures were fixed after OGD/PPADS treatment and fluorescent-migrating neuroblasts (green) were analyzed for the expression of DCX (red) 24 hours after the insult. *DiO co-cultures*. (C) Four day-old rat pups were i.v. injected with DiO and cultures prepared 8 hours later. Ipsilateral SVZ was plated in contact with contralateral cortex of the same injected animal (as indicated in the scheme). OGD was performed and migration of fluorescent neuroblasts (image 3, green) from the SVZ to the cortex was observed 24 later in the presence of 100 µM PPADS. Controls were performed in the presence of 1 mg/ml glucose (1, YFP/DCX merged). Co-cultures were fixed and fluorescent migrating neuroblasts were analyzed for the expression of DCX (2) at 10× (1–3) and 20× magnification (4, DiO/DCX merged).

In the first set of experiments, slices of fluorescent SVZ, obtained from YFP transgenic mice were cultivated in contact with wild-type cortex (Figure 4A). In this case, all neurons in the SVZ, both mature and immature, are fluorescent, whereas cortical cells do not express fluorescent proteins. Co-cultures were subjected to 30 minutes OGD in the presence or absence of PPADS and migration was observed during the first 24 hours after the insult. We observed a sporadic migration of fluorescent cells under control conditions which was slightly enhanced in slices treated with PPADS only. OGD induced a drastic reduction of fluorescent cells in the SVZ, but no migration at this time point was observed. OGD may affect the viability of precursor cells but not that of stem cells which remain in a resting state but are still able to generate new precursor cells (see also mathematical model below). Blockade of P2X receptors by PPADS not only sustained the survival of fluorescent cells, but also induced cell spreading from the SVZ into the cortex. Co-cultures were fixed 24 hours after OGD and subjected to DCX immunofluorescence to verify that fluorescent migrating cells were indeed neuroblasts (Figure 4B).

In the second set of co-culture experiments, we used SVZ labeled with the neuronal tracer DiO. Rat pups (4 day-old) were i.v. injected with DiO and 8 hours later sacrificed for organotypic culture preparation. Co-cultures were prepared with *labeled* ipsilateral SVZ and *unlabeled* contralateral cortex and fixed 24 hours after OGD (Figure 4C). Once again, PPADS was found to enhance the migration of fluorescent cells into the cortex under conditions of OGD; these migrating cells expressed DCX (Figure 4C_2–4_). In both experiments, the amount of migrating neuroblasts was found to be similar to that found in Figures 1–3 (data not shown).

### Factors that modulate neuroblast activation

**Figure 5:**
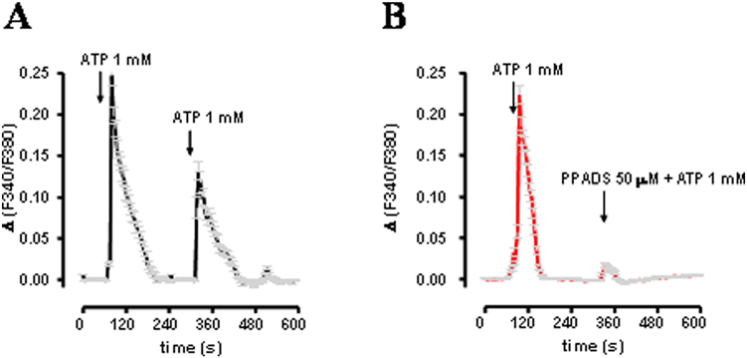
Cells from the SVZ respond to ATP by permitting extracellular calcium entry. (A) Neurospheres cultivated from the SVZ were used to measure ATP-mediated calcium influx. Neurospheres under control conditions were stimulated with 1 mM ATP, washed and re-stimulated in order to evaluate receptor desensitization. (B) PPADS efficiently blocks ATP-mediated calcium influx.

In order to demonstrate that the SVZ harbors cells which are sensitive to extracellular ATP, primary neurospheres obtained from SVZ disgregation were loaded with the fluorescent calcium indicator Fura-2 AM. The response of cells to a range of concentrations of extracellular ATP (from 100 µM to 5 mM; data not shown) was assayed. Neurospheres which contain both types of cells present in the SVZ (stem and progenitor cells), responded to bath applied 1 mM ATP (range concentration that resemble toxicity conditions) with an influx of extracellular calcium (Figure 5A). Calcium entry was specifically blocked by 50 µM PPADS - a lower concentration more accessible in neurospheres [28] - as shown in Figure 5B.

Involvement of extracellular ATP in modulating neuroblast activation was further confirmed by P2X_1–7_ ionotropic receptor subunit immunofluorescence, accompanied by counterstaining with DCX in the SVZ and cortex, under different experimental conditions. Of all the P2X subunits, only P2X_6_ and P2X_7_ colocalized with DCX in the SVZ (Tab. 2). OGD induced the expression of P2X_1_ and P2X_6_ in cortical neuroblasts, while inhibiting P2X_6_ expression in the SVZ. In contrast, expression of neuroblast P2X_7_ remained constant in the SVZ, under the different experimental conditions. PPADS added during OGD inhibited the expression of P2X_1_ and P2X_6_, but not P2X_7_.

**Table 2:** The expression of a subset of P2X receptors is modulated after OGD.

	CTRL	CTRL	OGD	OGD	OGD/PPADS	OGD/PPADS
	*SVZ*	*CX*	*SVZ*	*CX*	*SVZ*	*CX*
P2X1	−	−	−	+	−	−
P2X2	−	−	−	−	−	−
P2X3	−	−	−	−	−	−
P2X4	−	−	−	−	−	−
P2X5	−	−	−	−	−	−
P2X6	+	−	−	+	−	−
P2X7	+	−	+	−	+	−

P2X receptor subunit expression in neuroblasts during OGD was analyzed in organotypic cultures at 10 DIV. Slices were subjected to 30 min OGD in the presence or absence of PPADS and fixed 24 hours later. Co-expression of DCX with P2X receptor subunits was assessed by confocal analysis both in the SVZ and in the cortex under different experimental conditions. Abbreviations: CTRL, control consisting of 1 mg/ml glucose; OGD, oxygen and glucose deprivation; OGD/PPADS, OGD in the presence of 100 µM PPADS.

**Figure 6:**
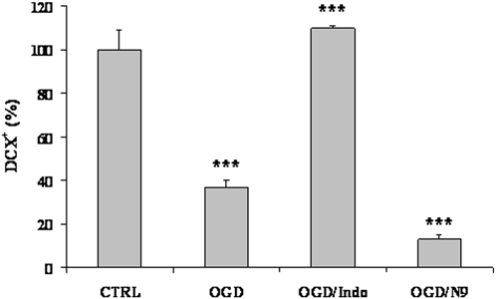
Microglial activation negatively affects neuroblast migration into the cortex. Organotypic cultures were co-cultivated with the microglial cell line N9 or treated with 10 µM indomethacine (OGD/Indo) during OGD. Cultures were fixed 24 hours later and DCX expressing cells were counted in the cortex and measured as a percentage of the control condition (1 mg/ml glucose). Counts in cells/mm2 represent means±SEM (n = 3).

In our attempt to model OGD-induced neuroblast activation, we took into account other factors which can modulate, positively or negatively, migration from the SVZ to the cortex. The role of microglial activation in neurogenesis after ischemia is currently strongly debated. Here, we treated organotypic culture with 10 µM indomethacine during OGD [16] and counted DCX^+^ neuroblasts in the cortex (Figure 6). We observed a reduction of approximately 60% in the number of DCX^+^ cells in the cortex 24 hours after OGD. Blocking microglial activation by indomethacine completely abrogated this effect. Furthermore, co-cultivating organotypic cultures with the N9 microglial cell line and subjecting these co-cultures to OGD drastically reduced the number of DCX^+^ cells in the cortex, further corroborating the idea that microglia activated by OGD negatively modulate neuroblast migration.

**Figure 7:**
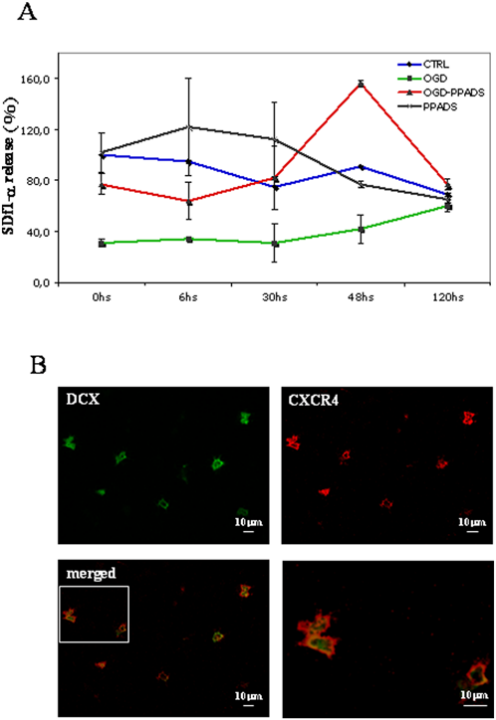
The SDf-1α chemokine as a guidance cue in OGD-induced neuronal migration. Cortex/SVZ/striatum cultures were subjected to 30 minutes of OGD in the presence or absence of 100 µM PPADS. PPADS was added during and after OGD or, as a control, alone. (A) Conditioned media were collected at different time points after OGD and examined by ELISA. SDf-1αrelease was measured as a percentage with respect to the control condition (1 mg/ml glucose). Counts represent means±SEM (n = 3). (B) DCX (green) and the CXCR4 receptor (red) were detected by double immunofluorescence. Merged capture is shown at two different magnifications. Double positive cells were photographed in cortical areas following OGD/PPADS-mediated migration.

Having identified factors which negatively regulate neuroblast activation, we searched for other factors which positively modulated neuroblast migration. It is well known that the migration of precursor cells is sustained by different chemokines, including SDf-1α. Thus, we measured the release of SDf-1α in organotypic cultures at different time points after OGD by ELISA (Figure 7A). We found that OGD induced a 70% decrease in the levels of SDf-1α early after OGD; these decreased levels remained low even 48 hours after the insult. Levels slightly increased only 5 days after OGD. P2X receptor antagonism by 100 µM PPADS during OGD led to a peak in SDf-1α release 48 hours after the insult (156% with respect to control); levels relapsed to control values 3 days later (77%). Curiously, PPADS administered in the presence of glucose (control condition) enhanced SDf-1α release for more than 30 hours. The keen reader will have noticed that SDf-1α measurements are out of phase by almost 24 h with respect to DCX counting data (peak in Figure 1A occurs at 24 h). This may be an artifact due to delayed solubilization of SDf-1α from tissue to the medium through membrane. To finally confirm that neuroblasts are migrating by a chemo-attractant gradient mechanism during PPADS treatment in OGD, we performed fluorescent double immunohistochemistry for the CXCR4 receptor (which is the receptor for SDf-1α) and DCX. As shown in Figure 7B, migrating neuroblasts expressed the CXCR4 receptor, supporting the assumption that SDf-1α gradients participate as guidance cues in neuroblast migration.

### Mathematical model of neuroblast activity induced by OGD

We have constructed a simple mathematical model with minimal number of parameters able to reproduce the neuroblast activity induced by OGD, both in the absence and presence of PPADS. It is important to note that by “minimal” we mean that removing a *single reaction* term in any of the equations causes the disruption of the qualitative adherence of the resulting dynamics with the experimental data set. The model is based on partial differential equations and involves the *balance*, *mass-action* and *Fick laws* [29] as its main biological assumptions. Our primary concern in building a reliable model was to express the assumptions in quantitative terms in order to isolate *in silico* the contribution to the dynamics of the main components of the model.

The spatial extension of the model is equivalent to an organotypic slice. The size of the germinative area and that of the ischemic area is ideally represented as regular and homogeneous (see Table S1 for Space-time parameters used in the simulations). The following assumptions were made (also listed in Table 3):

**Table 3:** Model reactions.

Reactions	Comments
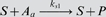	DCX^+^ generation
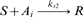	Inhibition of S by high concentration of ATP
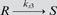	Resting S become active
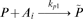	Inhibition of DCX^+^
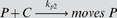	Movement by chemotaxis
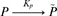	Death by aging
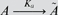	Death by aging
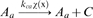	SDF-1α generation
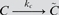	Death by aging

Model reactions summarizing the model assumptions. S, R, P, C, and A are the actors of the model (the tilde sign above the letter indicate a dead entity). A more detailed description of the mathematical model can be found in the Supplementary materials. Abbreviations: stem cells (S); precursors cells (P); basal concentration of ATP (A_a_); resting stem cells (R); high concentrations of ATP (A_i_); SDf-1α (C).

- Stem cells (S) generate precursors (P); the basal concentration of ATP (indicated by A_a_) contributes to their physiological functions;
- Resting stem cells (R) are temporarily inhibited by the presence of high concentrations of ATP (indicated by A_i_) released after in vitro ischemia;
- R cells become active after a certain delay;
- Precursor (P) cells die after in vitro ischemia in the presence of high concentrations of ATP;
- The chemokine SDf-1α (indicated by C) is released into the medium after ischemia;
- P cells follow the gradient of SDf-1α, hence migrating from the SVZ to the ischemic cortex.

The expected dynamics of the mathematical model of neuroblast activity after OGD can be summarized as follows: the ischemic event triggers the generation of a gradient of chemicals (i.e. signals) from the ischemic to the germinative area. The activation of stem cells, the generation of precursors and their migration to the ischemic area ensue. According to the different coefficients of the modeling equations, precursors can cover short or long distances to the ischemic area.

To actually observe the dynamics of the model equations (see Table S2 for constant parameters used in the simulation), we set up a computer simulation (for further details see Text S1, Figure S3, S4, S5, and Video S2, S3). The simulation reveals neuroblast activity which is consistent with both i) proliferation and migration of proliferating cells into the damaged areas in the absence of PPADS, and ii) earlier neuroblast migration followed by late proliferation in the presence of PPADS.

**Figure 8:**
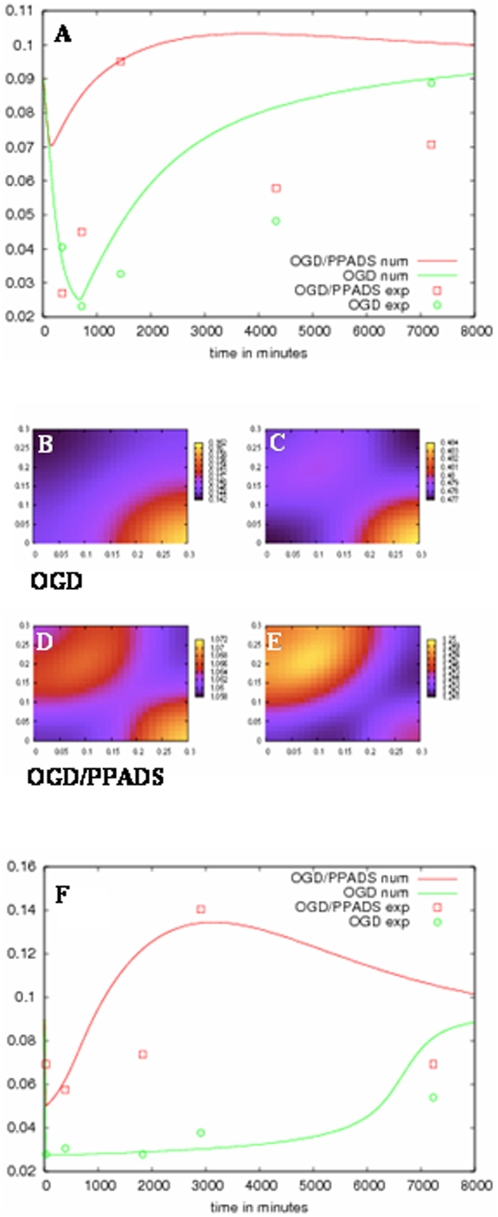
Modeled density of DCX+ and concentration of SDf-1α. (A) DCX+ density expressed as cells/mm2 as a function of time expressed in minutes after OGD. (B) Their spatial distribution in the slices under different conditions (OGD, panels B and C, and OGD with PPADS, panels D and E) at 12 (panels B and D) and 24 (panels C and E) hours after treatment. F) Concentration of SDf-1α as a function of time. Lines represent experimental data whereas dotted lines show the result of the mathematical model. Error percentage is calculated upon error bars expressed in Figure 1.

The overall picture is quite consistent with our biological observations. In particular, Figure 8B–8E shows the concentration of precursor cells at 12 and 24 hours both in the absence and presence of PPADS (see also Figure S5 and Video S2, S3). Our experimental data (Figure 1) is qualitatively recapitulated by the model's dynamics (Figure 8A). Shortly after OGD (6–24 hours), there is a reduction in the proliferative and migratory activity of precursors, whereas later (2 to 5 days after the insult), these cells start to proliferate and migrate into the damaged cortex. At the same time, a moderate increase in the level of SDf-1α is observed (Figure 8F).

In the presence of PPADS, the picture is different since much more SDf-1α is readily produced (within about 1 to 2 days after the insult) and consequently, neurogenic migration and successive proliferation occurs earlier and is more pronounced.

## Discussion

Modulation of endogenous neurogenesis following ischemia or other conditions of neuronal failure by pharmacological treatment represents a potential clinical tool with the valuable characteristics of not-invasiveness and efficiency. Current pharmacological treatments for ischemia are devoted to reestablishing vascularization and even anticoagulants and thrombolytics are used to prevent further attacks [30]. However, both approaches are merely palliative, and offer no promise of neuronal regeneration.

Over the last decade, which has seen remarkable progress in the area of stem cell research, cell therapy by embryonic or adult stem cell transplantation has been proposed as an alternative to pharmacological treatment, in order to regenerate lost neurons. Currently, cell therapy by autologous or heterologous stem cell transplantation seems to have been unsuccessful in this regard, due to the invasive nature of the treatment, cell rejection, death of transplanted cells and even ethical issues [31], [32]. In contrast, the induction of endogenous neurogenesis is a method which is free of the above-mentioned problems associated with pharmacological treatment and cell transplantation (for a review, see 33). Neurogenesis does physiologically occur after different brain diseases, but it has to be enhanced in order to definitively restore lost neuronal functions. It is well established that neurogenesis from cell birth to differentiation and finally to functional integration is modulated by molecular signals and influenced by both the micro and macro environment [34].

Neurorestorative strategies have two principal milestones: 1) research into novel mechanisms and new molecules involved in sustaining adult neurogenesis and 2) the identification of general rules which govern the behavior of all cell types involved in the regenerative machinery (stem cells, progenitor cells, newborn cells, and their responses to attractant and repelling molecules). One of the principal objectives of the multidisciplinary research reported herein was to develop a mathematical model on the basis of biological observations of adult neurogenesis induced by OGD. Several principles have emerged from our studies.

### Culture model

Organotypic cultures from different brain and CNS regions have become instrumental for the study of neurodegeneration following a variety of brain diseases [35], [36]. This culture model is also quite versatile, since electrophysiology as well as immunofluorescence techniques can be applied [37], [38]. It must be noted that organotypic slices, like any in vitro model, can give rise to artifacts and cellular reactions that are not seen in *in vivo* models. Upon preparation, gliosis involving astrocyte and microglia proliferation is observed [39]. Also, the three-dimensional structure is slightly remodeled (e.g., in our model, the area of the SVZ observed in culture is larger than that observed in vivo). The model reported herein is to our knowledge the first *in vitro* model representing tridimensionally the situation which occurs *in vivo* between a neurogenic region (SVZ) and a target region after a brain insult. Tridimensionality is completed by the presence of the *corpus callosum* which links the SVZ, cortex and striatum, mimicking the rostral migratory stream for precursor migration. Thus, this tissue model contains all of the fundamental elements necessary to recapitulate neuroblast activation and neurogenic responses. Moreover, through the fields-based, organotypic cultures can provide a template for the development of computer simulation, a feature which is not feasible with any other cellular models or in vivo.

### Dynamics

Genesis of newborn neurons was first described by Alvarez-Buylla [1], [40] and involved migration of cells from the adult SVZ into the olfactory bulb. Stem and progenitor cells differentiate through developmental stages with distinctive molecular and morphological properties [41], [42]. The characterization of a series of markers expressed at different stages of development (e.g. DCX for neuroblasts- 21, 43, 44), together with live experiments [21], helped to elucidate the dynamics of neurogenesis induced by OGD. In rodents, neurogenesis induced by global or focal ischemia is basically characterized by i) enhanced progenitor proliferation ii) migration of proliferating cells into damaged areas and iii) differentiation. Most studies have reported an increase in proliferation at 7–10 days, with persisting cell survival and differentiation for 2–3 weeks. It is evident that a large latent period exists between neuronal damage and neurogenesis activation. This period turns out to be an essential window of therapeutic opportunity for patient recovery and neuronal activity. Our group, as well as others, has demonstrated how damaged and neurogenic regions communicate with each other during an ischemic insult by molecular and diffusible factors [15], [23]. Gradients of molecules are generated and modulated by molecular mechanisms, thereby establishing a positive and negative environment, which in turn facilitates or delays proliferation, migration and differentiation. Our strategy to enhance neurogenesis towards neuronal replacement and recovery intends to shift this balance toward a more salutary environment, while minimizing negative factors.

One factor which contributes to impeding neurogenesis is high levels of extracellular ATP. Several features suggest that extracellular ATP plays a relevant role in modulating neurogenesis after OGD: i) massive amounts of ATP released following metabolism impairment have been shown to be neurotoxic [16], [24], [25]; ii) calcium signaling through purinergic P2X-Y receptors [45] regulates proliferation and differentiation of several stem cells types including muscle, bone marrow and neural stem cells [46], [47]; iii) purinergic signaling regulates neural progenitor expansion and corticogenesis under physiological condition [48] and iv) ectonucleotidases are functionally expressed on progenitor cells in postnatal and adult neurogenic zones [49]. Our model reproduces previously published data in which neurogenesis is transiently activated after OGD with a peak of DCX^+^ cells observed in the damaged cortex, 5 days after the insult. The lowest number of neuroblasts was found between 6 and 24 hours after OGD which is considered the latent period between neuronal damage and neurogenesis activation. Using our mathematical model, we can predict the consequences of pharmacological modulation of purinergic signaling. Several P2X receptors are expressed in the SVZ. Furthermore, these cells are ATP-sensitive and respond by permitting the influx of extracellular calcium. This reinforces the choice to use PPADS and TNP-ATP to block purinergic signaling through P2X receptors. Depending on the concentration of ATP in the extracellular milieu, it can act by sustaining stem cell proliferation (A_a_ in Tab. 3 of mathematical model), by leaving stem and progenitor cells in a resting state (A_i_ in Tab. 3) or by killing SVZ cells and neurons (excitotoxicity). These observations support the data published by Stafford and colleagues [50] on primary neurosphere cultures and highlight the critical role of extracellular ATP in modulating both neuronal survival and neurogenesis activation.

Blocking purinergic signaling allowed us to uncover other components of the dynamics of neurogenesis induction. Thus, in the absence of purinergic signaling (inhibited by P2X antagonists), the system responds to the ischemic insult with an SOS response. In fact, shortly after the insult (24 hours), a subpopulation of neuroblasts is liberated and rapidly recruited into the damaged area. In this response, proliferation is exhibited only five days after OGD. Experiments with AraC and neuroblast migration observed in co-culture exclude an activation of resident cortical neuroblasts. We hypothesize the presence of two neuroblast subpopulations in the SVZ. One can be rapidly recruited by halting proliferation and migrating into the damaged cortex. The second, already described elsewhere, is further activated and migrates only when the extracellular environment is buffered from toxic molecules. Analysis of SDf-1α release would also suggest that the two proposed neuroblast populations can migrate under different chemical/cellular mechanisms. In fact, after P2X modulation (by PPADS blockade), neuroblasts migrate under the influence of an intense SDf-1α signal (Figure 7, OGD/PPADS at 48 hours), whereas in the absence of PPADS, neuroblasts migrated (and proliferated) 5 days after OGD under the influence of low levels of released SDf-1α (Figure 7, OGD at 5 days).

Another point to be considered is brain inflammation which invariably occurs after ischemia. There has been a great debate about the role of inflammation and cytokine release after brain insult [51]. For example, infiltration of activated polymorphonuclear neutrophils into the injured parenchyma and the activation of microglia are playing an important role in the pathology of cerebral ischemia. Activated polymorphonuclear neutrophils can exacerbate brain damage by release of oxygen radicals or proinflammatory cytokine, whereas more debated is the role of microglia activation. Depending on the cytokine released and time of action, microglia activation can suppress or promote cell repair. More interesting became the role of neural stem cells as immunomodulators beside of repairing brain damage. There are several evidences that transplanted stem cells (after brain ischemia) can release factors acting as neuroprotectant (for a review see [14]). A neural stem cell-mediated peripheral immunomodulatory effect was postulated, through the modulation of macrophage-related TNF-α release.

Here, we have demonstrated that when inflammation is intense and additionally accompanied by other factors (such as high levels of extracellular ATP), it can impede neurogenesis.

### Functional implications

Our data show that in the face of massive neuronal loss, the rate of neuroblast migration is low (70–80 cells/mm^2^). This is in agreement with data previously published elsewhere (i.e. [52]). However, the functional consequences of this neurogenesis response remain unclear. In line with Lee and colleagues [53], we propose here some general mechanisms which may underlie the behavior of neural stem cells during adult neurogenesis. These researchers studied the effects of stem cell transplantation in a neurogenetic degenerative disease called Sandhoff's disease. Only a small percentage of neural stem cells transplanted in an animal model of the disease (Hexb^−/−^) differentiated into neurons. Moreover, disease symptoms and animal death were delayed, inflammation was reduced or immunosuppression was required. The authors discussed that although neuronal network replacement still receives most attention, this may be complemented by other stem cell function. In line with this work, it seems possible that adult stem and progenitor cells may act as “chaperone cells” by supporting the physiology of rested and damaged neurons; thus, they may sustain or preserve established circuitry, rather than attempting to reconstruct new connections, or participating in the reestablishment and buffering of the local environment.

We decided to use neuroblasts as an index of neurogenesis activation which was required for the development of the mathematical model. Nevertheless, further studies need to be done to investigate the fate of migrated neuroblasts (neuronal differentiation, integration into pre-existing networks, apoptosis), and to characterize and isolate the proposed neuroblast subpopulations. The mathematical model presented here should be of use for other studies of the functionality of adult neurogenesis. Numerical simulations can be useful to predict short and long-term consequences of using parameters and conditions in real experiments. Indeed, when there is no applicable animal model, computer modelling is often the only way to obtain new knowledge. The model we constructed here reproduces the qualitative behaviour of the system fulfilling the goal with the minimal parameters. However, a better fitting of the experimental data could be achieved by using a more sophisticated mathematical model justified only by a richer experimental data set.

## Supporting Information

Text S1 Materials and methods for organotypic cultures and mathematical model.
(0.14 MB DOC)

Figure S1 Characterization of cortex/SVZ/striatum organotypic cultures: the SVZ. Organotypic cultures at 10 DIV were primed in culture with 20 µM BrdU and fixed 2 hours later. BrdU (which labels proliferating cells) is shown in green and DCX (a neuroblast marker) in red in the SVZ. Bars represent 100 µm and 50 µm. Abbreviations: cx, cortex; cc, corpus callosum; SVZ, subventricular zone; str, striatum.
(0.13 MB TIF)

Figure S2 Characterization of cortex/SVZ/striatum organotypic cultures: the cortex. Organotypic cultures at 10 DIV were infected with pLVTHM-GFP and pictures of the cortex were taken with 10× (A) and 63× (B) magnification. (C) 3D reconstruction performed by LSM1 software after Z stack acquisition.
(0.10 MB TIF)

Figure S3 Model domains: cortex (in red) and subventricular zone (in green).
(0.04 MB TIF)

Figure S4 Activation/inhibition modalities of ATP. A low concentration of ATP is represented on the left side of the curve (activation response, Aa) and a high concentration is on the right (inhibition response, Ai).
(0.05 MB TIF)

Figure S5 Pharmacodynamics of PPADS.
(0.04 MB TIF)

Table S1 Space-time parameters used in the simulations.
(0.03 MB DOC)

Table S2 Constant parameters used in the simulations. Symbols c, t and s represent cells, time (in minutes), and spatial (in centimeters) unity, respectively.
(0.09 MB DOC)

Video S1 Ciliated cells in the SVZ of organotypic cultures. This organotypic culture at 10 DIV was recorded at 20× magnification with 190 frames of 10 milliseconds of delay, for a total length of 10 seconds.
(4.28 MB MOV)

Video S2 Computer simulation of neuroblast migration in the absence of PPADS. The video illustrates a representation of neuroblast migration (by means of counted DCX cells) from the SVZ to the cortex during 120 hours after OGD. SVZ is represented at the bottom right and the cortex at the top left of the video. The density of neuroblasts is represented by a colorimetric scale ranging from dark blue (low density) to red (high density) (see also Fig. 8B–8E).
(0.27 MB MOV)

Video S3 Computer simulation of neuroblast migration in the presence of PPADS. The video shows a representation of neuroblast migration (represented in terms of counted DCX cells) from the SVZ to the cortex during 120 hours after OGD in the presence of 100 µM PPADS. The SVZ is represented at the bottom right, and the cortex at the top left of the video. The density of neuroblasts is represented by a colorimetric scale ranging from dark blue (low density) to red (high density) (see also Fig. 8B–8E).
(0.32 MB MOV)
