# Supplementary material for: A Model of Ischemia-Induced Neuroblast Activation in the Adult Subventricular Zone
Source: PLoS One. 2009 Apr 23;4(4):e5278. doi: 10.1371/journal.pone.0005278 (PMC2669296; doi:10.1371/journal.pone.0005278)
Supplement: Text S1 — Materials and methods for organotypic cultures and mathematical model. (0.14 MB DOC) [file pone.0005278.s001.doc]

**MATERIALS AND METHODS**

**Organotypic culture measurements**

*The area of different brain regions* in the organotypic cultures was calculated with LSM Image software (Zeiss, Arese, Italy). Thus, the area of cortex and SVZ was 4.2 mm^2^ and 0.9 mm^2^ respectively. The number of neuroblasts (identified as DCX^+^ cells) was estimated by considering the area of a single neuroblast (approximately 15.71 μm^2^ [π (5 μm)^2^]), DCX fluorescence (by dpi intensity) and the area of the SVZ. The following formula was applied to estimate number of neuroblasts (n):

n = A_SVZ_/A_DCX_*[1/*K*_dpi_] where A_SVZ_ and A_DCX_ are the area of the SVZ and of a single neuroblast respectively and *K*_dpi_ is a constant given by the mean DCX intensity after immunofluorescence and normalized to the background of the area.

**Viral infections**

Viral particles were produced in human embryonic kidney 293T cells (ATCC, www.atcc.org). Briefly, 293T cells were seeded in high glucose DMEM containing 10% FBS. The lentiviral vector pLVTHM (Addgene plasmid 12247, www.addgene.org) containing the GFP reporter gene was transfected into the packaging cell line by calcium phosphate precipitation in the presence of 25 μM chloroquine (Sigma). Transductions were carried out in the presence of 16 μg/ml polybrene (Sigma). The concentrated viral particles were directly placed on top of organotypic cultures for 6 hours and then washed off with fresh medium and cultured for the indicated time.

### MATHEMATICAL MODEL

**Model without drug**

Let us consider a given region Q of the brain, here in two dimensional symmetry, including the cortex region and the SVZ (see figure S3 and table S1 for the used space-time dimensions). We introduce the main concentrations involved in the model:

$p=p(\mathbf{x},t)$, concentration of precursor (assumed as number of neuroblasts/mm^2^)

$c=c(\mathbf{x},t)$, concentration of SDf1-α,

$a=a(\mathbf{x},t)$, concentration of ATP,

$s=s(\mathbf{x},t)$, concentration of stem cells (active form-assumed as number of stem cells/mm^2^)

$r=r(\mathbf{x},t)$, concentration of stem cells (inactive/refractory form).

The model equations are:

The parameters used in the simulations are summarized in table S2. The other functions are described as follows. For the evolution of precursor concentration, we assumed a transport mechanism driven by SDf-1α**-**mediated chemotaxis. The transport field is proportional to the gradient of SDf-1α**,** the concentration of precursors and to the function$\Psi(c)$, which represents chemotactic sensitivity and describes the saturation effects on the receptors of precursors with respect to the sensitivity to the SDf-1α gradient. The source/sink terms in the precursor equations are modulated by the effect of ATP. More precisely, we introduce a function $f(a)$, which represents the two modalities of ATP as activator (A_a_, low concentration) and inhibitor (A_i_, high concentration). This single function determines A*_a_* and A*_i_*, but with positive or negative significance by means of $A_{a}=\left[f(a)\right]_{+}$and$A_{i}=\left[f(a)\right]$. The parameters which determine the functional form of $f(a)$ are given by$a_{0}$, which is the transition concentration of ATP between activation and inhibition of the system, and$a_{ofs}$, which is the offset value needed to reach the inhibitory regime. The functional form of $f(a)$ is as follows (see also figure S4):

$$f(a)=\left\{\begin{matrix} \frac{2a_{0}}{\pi}\sin\left(\frac{\pi}{2}\frac{a}{a_{0}}\right), & \text{if }0\leq a\leq a_{0}, \\ \frac{2a_{0}}{\pi a_{ofs}}\exp\left(a_{0}-a\right)\left(a_{0}+a_{ofs}-a\right), & \text{if }a_{0}<a\leq a_{0}+a_{ofs}, \\ -\frac{2a_{0}}{\pi}\sin\left(\frac{\pi}{2}\frac{a-a_{0}-a_{ofs}}{a_{0}}\right), & \text{if }a_{0}+a_{ofs}<a\leq2a_{0}+a_{ofs}, \\ -\frac{2a_{0}}{\pi}, & \text{if }a>2a_{0}+a_{ofs}. \end{matrix}\right.$$

We introduce the term

$$\tilde{k}_{p}=\frac{k_{s1}\int_{Q} s\left[f(a)\right]_{+}d\mathbf{x}+k_{p1}\int_{Q} p\left[f(a)\right]_{-}d\mathbf{x}}{p_{0}\left|Q\right|}$$

to guarantee a correct convergence of the system at equilibrium. For the concentration of ATP and SDf-1α, we assumed a diffusion mechanism, with diffusion coefficients $d_{c}$and $d_{a}$respectively. The function $\Phi(\mathbf{x})$ delineates the region of the cortex (see again figure S3): $\Phi(\mathbf{x})=\exp\left(-\frac{\left(\xi-c_{\xi}\right)^{2}}{2(\sigma_{x})^{2}}-\frac{\left(\eta-c_{\eta}\right)^{2}}{2(\sigma_{y})^{2}}\right)$ with

$\xi=x\cos(\pi/4)-y\sin(\pi/4)$,$\eta=x\sin(\pi/4)+y\cos(\pi/4)$, $c_{\xi}=c_{x}\cos(\pi/4)-c_{y}\cos(\pi/4)$,and$c_{\eta}=c_{x}\sin(\pi/4)+c_{y}\cos(\pi/4)$.

The function describes the presence of oxygen,.

Finally, the evolution of stem cells and their inactive form is driven by a simple conservative mechanism, with no movement of cells which is also driven by the inhibitor phase of ATP.

**Model with drug**

As previously stated, pharmacological blockade of extracellular ATP receptors by PPADS during and after the insult unmasks the tendency of the SVZ to send a rapid SOS signal represented by the migration of neuroblasts which previously did not proliferate. To calculate the effect of PPADS on neuroblast dynamics, we considered this effect as follows. The pharmacodynamics is modeled by means of the following function (see figure S5):

$g(t)=\frac{Ge^{2}}{4\tau^{2}}t^{2}e^{-t/\tau}$ that has a maximum (G) at time $t^{*}=2\tau$.

The effect of the drug is modeled by changing the threshold value $a_{0}$ of the function $f(a)$, and the asymptotic concentration of SDf1-alpha $\bar{c}$ as follows: $a_{0}$ becomes $a_{0}+g_{a}(t)$ and $\bar{c}$ becomes $\bar{c}+g_{c}(t)$, where the functions $g_{a}$ and $g_{c}$

are just the function $g$with the parameter *G* calibrated again experimental tests (see table S2). Moreover we change the death rate $k_{co}$of SDf1-α during ischemia, to model the assumption that the drug protects the cells involved in SDf1-α degradation/uptake: $k_{co}$becomes $\alpha k_{co}$with$0<\alpha<1$. The equation for SDf1-α then becomes

$\partial_{t}c=d_{c}\Delta c+\frac{e^{-cs/c}}{\tau_{r}}(\bar{c}+g_{c}(t)-c)\Phi(x)-\alpha k_{co}(1-O(t))c$.

*Numerical simulations*

We first evolved the system with $O(t)=1$ in order to obtain the asymptotic stationary state. This constitutes the initial condition (barred variables, i.e.,$\bar{c}$). Then, we evolved the system simulating the ischemic event by setting$O(t)=0$. The parameters used are reported in table S1 and table S2.
