# Supplementary figures and images for: A Model of Ischemia-Induced Neuroblast Activation in the Adult Subventricular Zone
Source: PLoS One. 2009 Apr 23;4(4):e5278. doi: 10.1371/journal.pone.0005278 (PMC2669296; doi:10.1371/journal.pone.0005278)

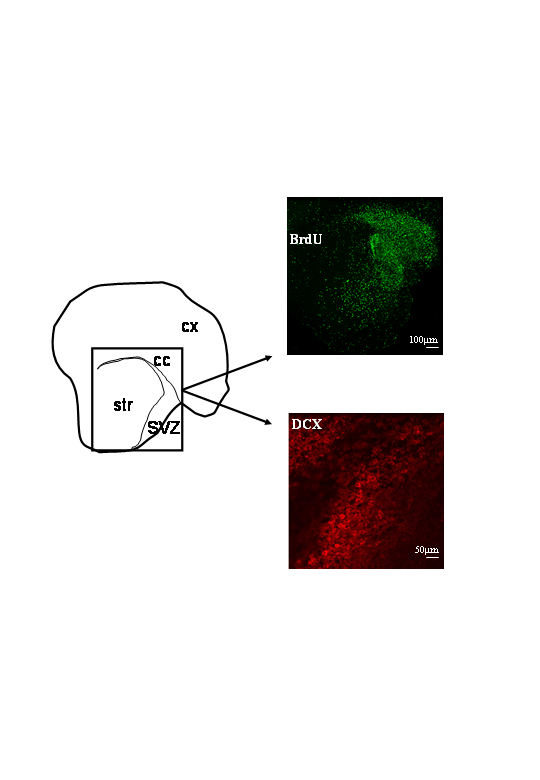

Supplement: Figure S1 — Characterization of cortex/SVZ/striatum organotypic cultures: the SVZ. Organotypic cultures at 10 DIV were primed in culture with 20 µM BrdU and fixed 2 hours later. BrdU (which labels proliferating cells) is shown in green and DCX (a neuroblast marker) in red in the SVZ. Bars represent 100 µm and 50 µm. Abbreviations: cx, cortex; cc, corpus callosum; SVZ, subventricular zone; str, striatum. (0.13 MB TIF) [file pone.0005278.s002.tif]

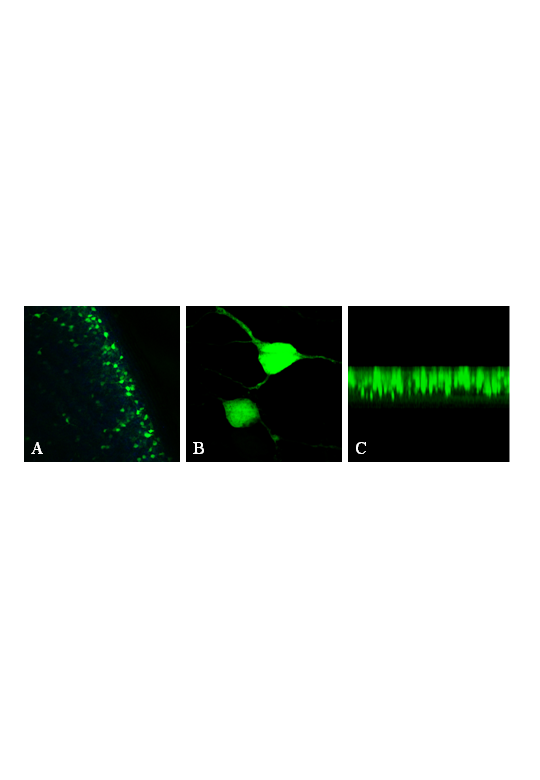

Supplement: Figure S2 — Characterization of cortex/SVZ/striatum organotypic cultures: the cortex. Organotypic cultures at 10 DIV were infected with pLVTHM-GFP and pictures of the cortex were taken with 10× (A) and 63× (B) magnification. (C) 3D reconstruction performed by LSM1 software after Z stack acquisition. (0.10 MB TIF) [file pone.0005278.s003.tif]

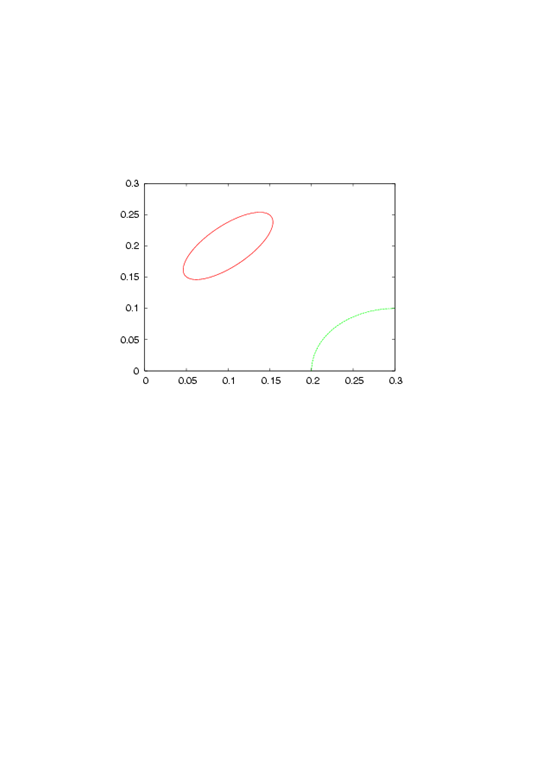

Supplement: Figure S3 — Model domains: cortex (in red) and subventricular zone (in green). (0.04 MB TIF) [file pone.0005278.s004.tif]

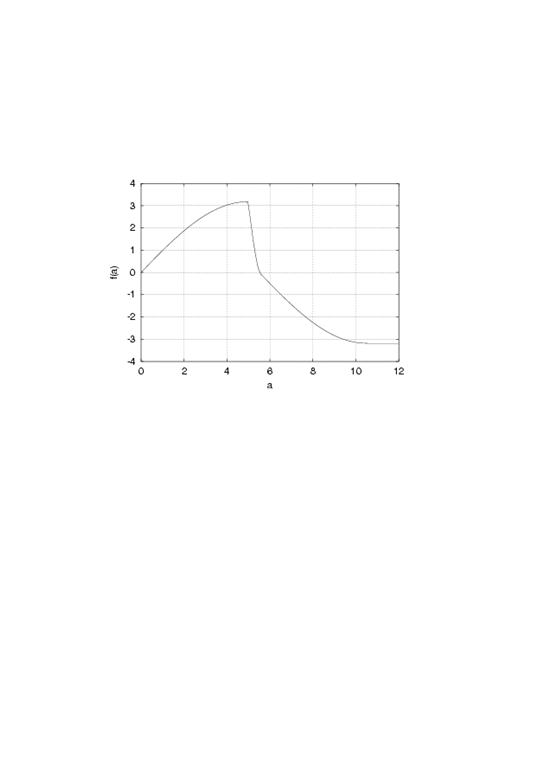

Supplement: Figure S4 — Activation/inhibition modalities of ATP. A low concentration of ATP is represented on the left side of the curve (activation response, Aa) and a high concentration is on the right (inhibition response, Ai). (0.05 MB TIF) [file pone.0005278.s005.tif]

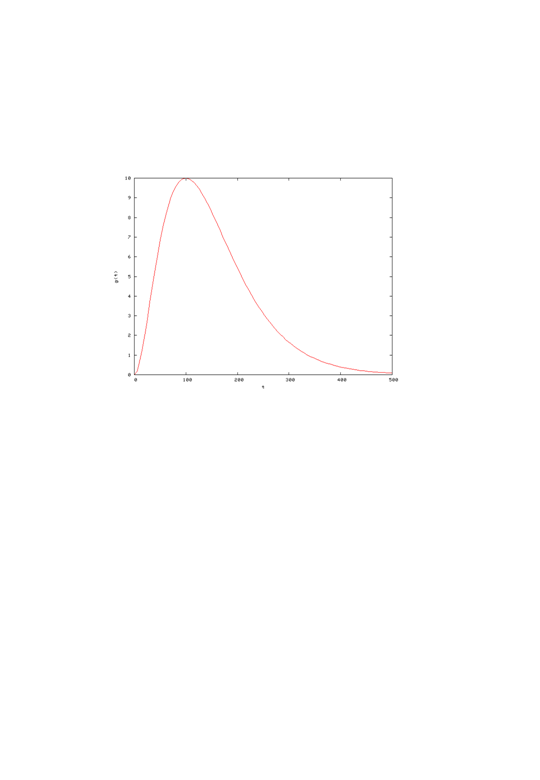

Supplement: Figure S5 — Pharmacodynamics of PPADS. (0.04 MB TIF) [file pone.0005278.s006.tif]
