# Supplementary material for: A Model of Ischemia-Induced Neuroblast Activation in the Adult Subventricular Zone
Source: PLoS One. 2009 Apr 23;4(4):e5278. doi: 10.1371/journal.pone.0005278 (PMC2669296; doi:10.1371/journal.pone.0005278)
Supplement: Table S1 — Space-time parameters used in the simulations. (0.03 MB DOC) [file pone.0005278.s007.doc]

| **Symbol** | **Description** | **Value** |
| --- | --- | --- |
| $L$ | Edge of the domain | 0.3 cm |
| $N$ | Number of grid points in x and y directions | 25 |
| $T$ | Final time | 8000 min (about 120 hours) |
| $r_{s}$ | Radius of the staminal region | 0.1 cm |
| $(c_{x},c_{y})$ | Centre of the cortex region | (0.1 cm, 0.2 cm) |
| $\sigma_{x},\sigma_{y}$ | Parameters of the cortex region | 0.02 cm, 0.05 cm |
