# Supplementary material for: A Model of Ischemia-Induced Neuroblast Activation in the Adult Subventricular Zone
Source: PLoS One. 2009 Apr 23;4(4):e5278. doi: 10.1371/journal.pone.0005278 (PMC2669296; doi:10.1371/journal.pone.0005278)
Supplement: Table S2 — Constant parameters used in the simulations. Symbols c, t and s represent cells, time (in minutes), and spatial (in centimeters) unity, respectively. (0.09 MB DOC) [file pone.0005278.s008.doc]

| **Symbol** | **Description** | **Value** | **Units** |
| --- | --- | --- | --- |
| $k_{p2}$ | Chemotactic coefficient of DCX | 0.0001 | cs^4^t^-1^ |
| $k_{s1}$ | Generation rate of DCX | 0.002 | c^-1^t^-1^ |
| $k_{p}$ | Degradation rate of DCX | 0.003 | t^-1^ |
| $k_{p1}$ | Inhibition rate of DCX | 0.003 | c^-1^t^-1^ |
| $d_{a}$ | Diffusion coefficient of ATP | 1e-4 | s^2^t^-1^ |
| $k_{a}$ | Degradation rate of ATP | 9e-4 | t^-1^ |
| $k_{ao}$ | Production rate of ATP due to OGD | 9e-4 | ct^-1^ |
| $a_{0}$ | Threshold concentration of ATP | 2.5 | c |
| $a_{ofs}$ | Offset concentration of ATP | 0.1 | c |
| $d_{c}$ | Diffusion coefficient of SDf1-α | 5e-5 | s^2^t^-1^ |
| $k_{co}$ | Degradation rate of SDf1-α due to OGD | 0.04 | t^-1^ |
| $c_{s}$ | Threshold concentration of SDf1-α | 2.6 | c |
| $\tau_{r}$ | Production rate of SDF1-α | 200 | t |
| $k_{s2}$ | Inhibition rate of stem cells | 0.01 | c^-1^t^-1^ |
| $k_{s3}$ | Escape rate from inhibition of stem cells | 0.05 | t^-1^ |
| $G_{a},\tau_{a}$ | Parameters for function $g$ for ATP threshold | 2, 360 min | c,t |
| $G_{c},\tau_{c}$ | Parameters for function $g$ for SDf1-α stationary concentration | 0.5, 1440 min | c,t |
| $\alpha$ | Parameters to model SDf1-α protection during OGD | 0.05 | dimensionless |
